# Ostensive-Cue Sensitive Learning and Exclusive Evaluation of Policies: A Solution for Measuring Contingency of Experiences for Social Developmental Robot

**DOI:** 10.3389/frobt.2019.00002

**Published:** 2019-01-29

**Authors:** Hamed Mahzoon, Yuichiro Yoshikawa, Hiroshi Ishiguro

**Affiliations:** Intelligent Robotics Laboratory, Department of System Innovation, Graduate School of Engineering Science, Osaka University, Osaka, Japan

**Keywords:** contingency evaluation, developmental robot, ostensive cue, human-robot interaction, joint attention

## Abstract

Joint attention related behaviors (JARBs) are some of the most important and basic cognitive functions for establishing successful communication in human interaction. It is learned gradually during the infant's developmental process, and enables the infant to purposefully improve his/her interaction with the others. To adopt such a developmental process for building an adaptive and social robot, previous studies proposed several contingency evaluation methods, by which an infant robot becomes able to sequentially learn some primary social skills. These skills included gaze following and social referencing, and could be acquired through interacting with a human caregiver model in a computer simulation. However, to implement such methods to a real-world robot, two major problems, that were not addressed in the previous research, have remained unresearched: (1) dependency of histogram of the observed events by the robot to each other, which increases the error of the internal calculation and consequently decreases the accuracy of contingency evaluation; and (2) unsynchronized teaching/learning phase of the teaching-caregiver and the learning-robot, which leads the robot and the caregiver not to understand the suitable timing for the learning and the teaching, respectively. In this paper, we address these two problems, and propose two algorithms in order to solve them: (1) exclusive evaluation of policies (XEP) for the former, and (2) ostensive-cue sensitive learning (OsL) for the latter. To show the effect of the proposed algorithms, we conducted a real-world human-robot interaction experiment with 48 subjects, and compared the performance of the learning robot with/without proposed algorithms. Our results show that adopting proposed algorithms improves the robot's performance in terms of learning efficiency, complexity of the learned behaviors, predictability of the robot, and even the result of the subjective evaluation of the participants about the intelligence of the robot as well as the quality of the interaction.

## 1. Introduction

Joint attention related behaviors (JARBs) include basic social skills, such as following the gaze of others, pointing, intention sharing, and social referencing. Humans gradually learn these social skills during their developmental process in infancy and childhood (Scaife and Bruner, 1975; Adamson, 1995; Corkum and Moore, 1995), and become able to establish interaction with others. Consequently, children become able to learn more social skills, such as language communication and mind reading (Moore and Dunham, 2014). The importance of JARBs in human infant development (Tomasello et al., 1995) has made it one of the most popular research topics in the fields of cognitive science and developmental psychology (Butterworth and Jarrett, 1991; Mundy et al., 2000; Tomasello, 2009).

Additionally, owing to the important role of such behaviors in achieving successful communication with humans, some robotic research has focused on the study of JARBs in the development of communicative robots (Imai et al., 2003; Breazeal, 2004; Kanda et al., 2004; Kaplan and Hafner, 2006).

On the other hand, in the field of developmental robotics, several studies based on synthetic approaches have tried to explore and/or reproduce the developmental process of the human infant, as well as to create autonomous developmental robots. See Asada et al. (2009) for a review of these efforts. Some of these research has been done on proposing learning mechanisms based on the intrinsic motivation of the robot that enables open-ended development (Oudeyer et al., 2007; Barto, 2013; Nehmzow et al., 2013), and some on dynamic Bayesian networks to evaluate the contingency of the observed events, which enables the robot to plan suitable action(s) to achieve its goal utilizing the evaluated contingency (Degris et al., 2006; Jonsson and Barto, 2007; Mugan and Kuipers, 2012).

Other studies (Nagai et al., 2003; Triesch et al., 2006) have tried to explain the developmental process of the JARBs of the human infant by using an infant robot. They have focused on the causality of the infant robot's observations, actions and consequent experiences during interaction with a human caregiver. They showed that learning of the causal sensorimotor mapping from gaze patterns of the caregiver to the motor commands of the robot lead the robot to acquire a primitive JARBs, such as gaze following. However, the robot had *a priori* knowledge of the set of sensory and motor variables to be associated in order to acquire such a sensorimotor mapping.

Sumioka et al. proposed an informational measure based on transfer entropy (Schreiber, 2000), by which the robot become able to automatically distinguish the set of sensory-motor variables for the sensorimotor mapping without such *a priori* knowledge (Sumioka et al., 2010). Additionally, their presented method could evaluate the contingency of a sequence of events, so that the robot became able to learn a sequence of sensorimotor mapping. The contingency of such sequence was defined as *contingency chain* (c-Chain). By using computer simulation, they showed that evaluating the c-Chains of the events led their infant robot model to learn JARBs consisted of sequences of actions, such as *social referencing* behavior. The social referencing was defined as looking back at the caregiver's face after producing the gaze-following behavior. Hereafter, we refer to robot's learned behavior as a *complex skill* if it consists of more than two sequences of actions (such as social referencing behavior), and otherwise refer to it as a *simple skill* (such as gaze-following behavior).

However, numerous time steps were required for the contingency evaluations of previous work (Sumioka et al., 2010), especially for complex skills, which resulted in the robot not being able to acquire complex skills in the real-world implementation (Sumioka et al., 2013). Mahzoon et al. (2016) proposed a new informational measure based on what they called *transfer information*, which enabled the local evaluation of the contingency among the variable values. They realized a fast contingency evaluation, even with a small number of sample data. They showed that their infant robot model could acquire simple and complex skills within short periods of interaction with the caregiver model, in a computer simulation environment.

Nevertheless, to implement the proposed method on a real-world robot, two basic issues are still remained: First, the synchronization problem of the robot's learning phase with the human caregiver's teaching phase in the real-world interaction was not considered. As a result, the efficiency of the learning process was decreased and therefore unexpectedly delayed. Although understanding and detecting the teaching phase of the human caregiver is not a simple issue, some research on “natural pedagogy” has reported the phenomena of teaching/learning timing of the human caregiver/infant (Csibra and Gergely, 2009) and addressed “ostensive cues” as the key signals of efficient teaching/learning in humans. In this paper, we propose a new algorithm for robot learning inspired by these phenomena, namely ostensive-cue sensitive learning (OsL), to overcome the synchronization problem. Second, there was overestimation of the contingencies related to actions/observations that occur simultaneously with the usage of a learned behavior. This is due to the confusion of the robot about the cause of the consequent event; the robot could not distinguish whether the reason for the event was the usage of the learned behavior or simply the previous atomic action/observation. To solve this problem, we propose another new algorithm, the exclusive evaluation of policies (XEP), following which the robot evaluates contingencies, so that the calculations related to the atomic variables are separated from those of the learned behaviors.

To evaluate the performance of each proposed algorithm in a real-world environment, we conducted human–robot interaction experiments under four conditions: (1) the previous method (Mahzoon et al., 2016), i.e., the robot uses neither of the proposed algorithms; (2) the robot uses only the OsL; (3) the robot uses only the XEP; and (4) the proposed method, i.e., the robot uses both the OsL and XEP. Each condition was consisted of 12 subject experiments, and each experiment was taken 800 time steps, i.e., approximately 40 min of interaction with the robot. The performances of the systems was compared in terms of the speed, coverage, and reliability of simple and complex skill acquisition.

In addition, as described in Moore and Dunham (2014) and Tomasello (2009), contingent and intelligent behavior of the infant “induces” the caregiver to change its behavior, and teach new concepts to the infant. This inherent tendency of the human caregiver leads to a potential for the open-ended learning and development of the infant, even an infant robot (Oudeyer et al., 2007). In our experiment, to evaluate if/how the human subjects feel regarding the infant robot's such intelligence, we conducted a subjective evaluation during the experiment. We asked the subjective opinion of the caregivers about the intelligence of the robot as well as the quality of the interaction. For this, we provided seven questions, each designed with a five-level Likert scale answer. To see the effect of the proposed algorithms on the subjective evaluation, we conducted a statistical analysis of the answers. The result of the analysis is discussed in section 4.5.

## 2. Problem Setting and Contingency Evaluation

### 2.1. Interaction Environment

**Figure 1:**
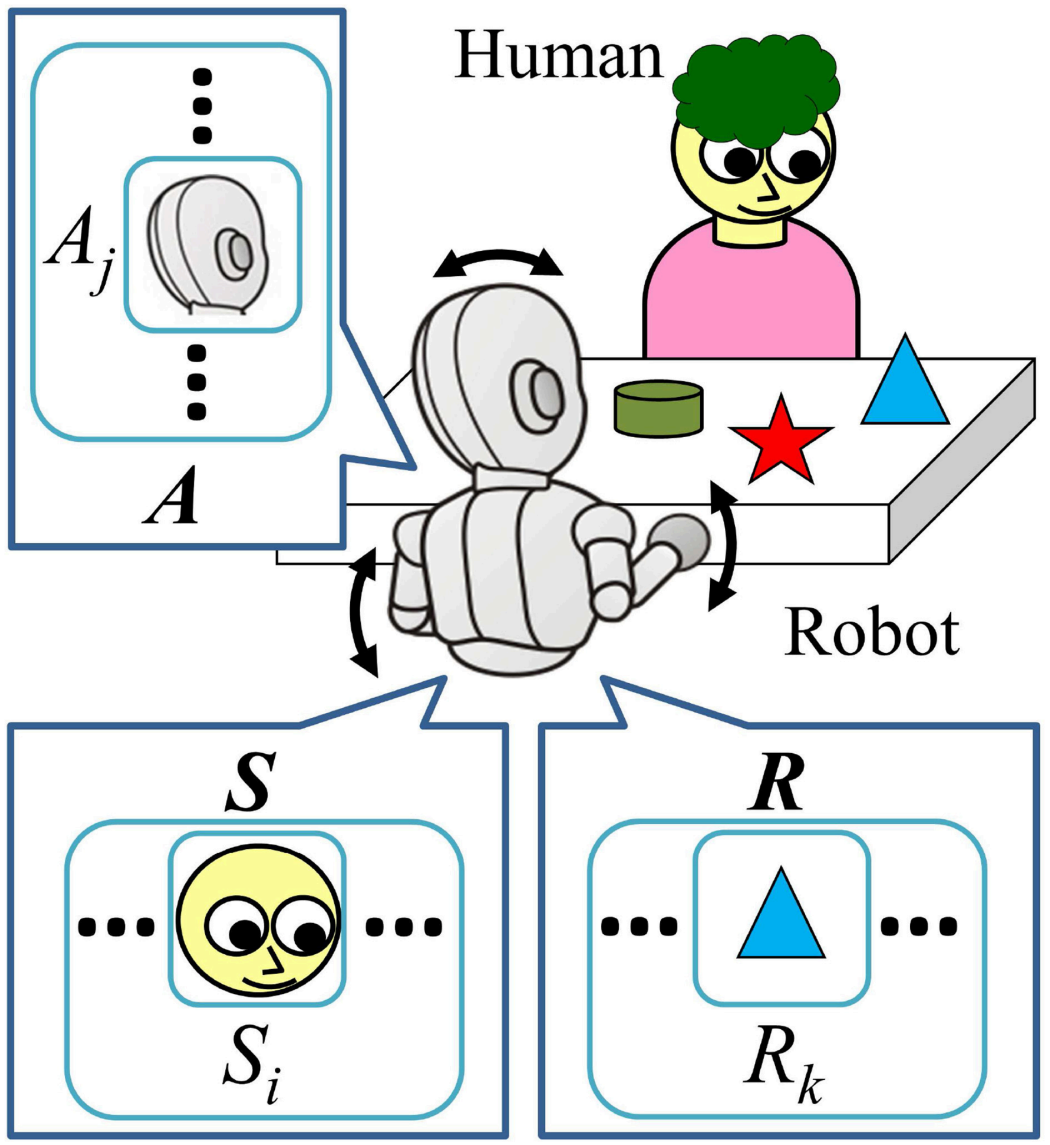
Problem setting of the face-to-face interaction of the robot with a human caregiver. They sit across a table, and there are some objects on the table. The robot can produce actions such as moving head and hands as illustrated with the arrows in the figure. During the interaction with the human, the sensory data, taken actions, and resultant observations are stored in the sensory, action, and resultant variables, respectively (***S***, ***A***, and ***R***).

A face-to-face interaction between a human caregiver and an (infant) robot is assumed as our experimental environment (Figure 1). There is a table between them and one or more objects are placed on the table. The human caregiver plays and interacts with the robot (based on their own strategy, if any) and can move the position of the objects on the table. The robot discretizes time. At each time step *t*, the robot observes the environment and stores the observed data in the sensory variables $S^{t}={\left(S_{1}^{t},S_{2}^{t},\cdots \;,S_{N_{S}}^{t}\right)}^{T}$, where *N*_*S*_ denotes the number of sensory variables. We also refer to these by “state variable” in this paper. After the observation, it sends action commands to its joints and saves them to the action variables $A^{t}={\left(A_{1}^{t},A_{2}^{t},\cdots \;,A_{N_{A}}^{t}\right)}^{T}$, where *N*_*A*_ denotes the number of action variables, which would be equal to the number of the joints of the robot. Next, the robot observes the result of the taken action, and saves the resultant observations to the resultant variables: $R^{t}={\left(R_{1}^{t},R_{2}^{t},\cdots \;,R_{N_{R}}^{t}\right)}^{T}$ for the values of the resultant observation before taking the action, and $R^{t+1}={\left(R_{1}^{t+1},R_{2}^{t+1},\cdots \;,R_{N_{R}}^{t+1}\right)}^{T}$ for after taking the action, where *N*_*R*_ denotes the number of the resultant variables. In the remainder of this section, we summarize and introduce the basic idea of the contingency evaluation mechanism of our previous work (Mahzoon et al., 2016).

### 2.2. Finding and Reproducing Contingency

Assume that in time step *t*, the robot observes $s_{i}^{t}$ and $r_{k}^{t}$, takes the action $a_{j}^{t}$, and as result, observes $r_{k}^{t+1}$; here, $s_{i}^{t}$, $a_{j}^{t}$, $r_{k}^{t}$, and $r_{k}^{t+1}$ indicate the values of the variables $S_{i}^{t}$, $A_{j}^{t}$, $R_{k}^{t}$, and $R_{k}^{t+1}$, respectively. The quaternion $e=\left(s_{i}^{t},a_{j}^{t},r_{k}^{t},r_{k}^{t+1}\right)$ represents such an experience of the robot, and is simply denoted as *experience* in this paper. An experience ***e*** contains information about “*when* $\left(s_{i}^{t}\right)$*, what to do* $\left(a_{j}^{t}\right)$*, for which transition* ($r_{k}^{t}$ to $r_{k}^{t+1}$).” During the interaction with the human, the robot evaluates the “contingency” of its experiences, which will be described later, and distinguishes the “contingent” ones. After finding the contingent experience(s), the robot tries to “reproduce” it by acquiring a suitable sensorimotor mapping that enables the robot to take suitable action $a_{j}^{t}$ in the specific state $s_{i}^{t}$ to reproduce the specific transition of $r_{k}^{t}$ to $r_{k}^{t+1}$. Inspired by previous works on human infant behaviors concerning the process of finding and reproducing interaction contingencies (Watson, 1972), even with a contingently responsive robot (Movellan and Watson, 2002), in our work, the ability to reproduce the contingency of an interaction is considered to be one of the most essential social skills for an interactional robot, which makes it able to interact properly with the interacting human.

To evaluate the contingency of the experiences, the robot updates and saves histograms of the values of the variables in each step of the interaction, and calculates the following probabilities. Assume there are two discrete-time stochastic processes *X* and *Y*, which can be approximated by stationary Markov processes. The transitions of the processes from time *t* to *t*+1 can be represented by the transition probabilities *p*(*x*^*t*+1^|*x*^*t*^) and *p*(*y*^*t*+1^|*y*^*t*^), where the notifications *x*^*t*^, *y*^*t*^ and *x*^*t*+1^, *y*^*t*+1^ indicate the values of the processes at times *t* and *t* + 1, respectively. The contribution of a specific value of process *Y*, such as *y*^*t*^, on the transition of the process *X* from a specific value such as *x*^*t*^ to a specific value *x*^*t*+1^ can be estimated using *transfer information* (Mahzoon et al., 2016):

(1) $$\begin{array}{l} I_{y\to x}=\log\frac{p\left(x^{t+1}|x^{t},y^{t}\right)}{p\left(x^{t+1}|x^{t}\right)}. \end{array}$$

For an experience ***e***, the transfer information can be adopted as follows to evaluate the contingency of the experience, i.e., the contribution of the action $a_{j}^{t}$ in state $s_{i}^{t}$ to the transition of $r_{k}^{t}$ to $r_{k}^{t+1}$, or in other words the joint contribution of the state and action in experience ***e***:

(2) $$\begin{array}{l} C_{J}\left(e\right)=I_{\left(s_{i},a_{j}\right)\to r_{k}}=\log\frac{p\left(r_{k}^{t+1}|s_{i}^{t},a_{j}^{t},r_{k}^{t}\right)}{p\left(r_{k}^{t+1}|r_{k}^{t}\right)}. \end{array}$$

Additionally, the single contributions of the state and action in experience ***e*** can be calculated as follows:

(3) $$\begin{array}{l} C_{S}\left(e\right)=I_{s_{i}\to r_{k}}=\log\frac{p\left(r_{k}^{t+1}|s_{i}^{t},r_{k}^{t}\right)}{p\left(r_{k}^{t+1}|r_{k}^{t}\right)}, \end{array}$$

(4) $$\begin{array}{l} C_{A}\left(e\right)=I_{a_{j}\to r_{k}}=\log\frac{p\left(r_{k}^{t+1}|a_{j}^{t},r_{k}^{t}\right)}{p\left(r_{k}^{t+1}|r_{k}^{t}\right)}. \end{array}$$

The purpose of the robot is to evaluate the joint contribution in experiences to know if the action $a_{j}^{t}$ in state $s_{i}^{t}$ specifically leads to the consistent result $r_{k}^{t+1}$, and acquire a sensorimotor mapping of $s_{i}^{t}$ to $a_{j}^{t}$. However, the value of Equation (2) can be also large when the value of the single contribution of either the state or action becomes large. Therefore, the joint contribution needs to be compared with the single contributions to distinguish the experiences in which the transition to $r_{k}^{t+1}$ is due to both $s_{i}^{t}$ and $a_{j}^{t}$, and not simply one of them. It can be estimated as follows:

(5) C˜SJ(e)=CJ(e)−CS(e)                =logp(rkt+1|sit,ajt,rkt)p(rkt+1|sit,rkt),

(6) C˜AJ(e)=CJ(e)−CA(e)                =logp(rkt+1|sit,ajt,rkt)p(rkt+1|ajt,rkt),

where C˜SJ(e) and C˜AJ(e) compare the joint contribution with the single contribution of the state and action, respectively. Finally, the measure named *synergistic contribution of contingencies* (ScC) is proposed as follows to distinguish the “*contingent*” experiences, i.e., the experiences in which the combination of the state and the action is the cause of the transition, but not either of them is individually the cause:

(7) C˜J(e)=min{C˜SJ(e),C˜AJ(e)}.

When the value of ${\tilde{C}}_{J}\left(e\right)$ of a specific experience ***e*** becomes larger than a specific threshold *C*_*T*_ for a predefined duration, such as θ time steps, the robot distinguishes it as a contingent experience (or simply, a contingency) and acquires the sensorimotor mapping $\left(s_{i}^{t},a_{j}^{t}\right)$. Then, it starts to “reproduce" the found contingency by “using” the acquired sensorimotor mapping. The sensorimotor mapping learned based on the experience ***e*** is denoted as the policy π. During interaction with the human, the robot may acquire several different policies. Note that θ is a parameter to determine how carefully the observed contingency is judged to be stable.

### 2.3. Evaluating the Contingency Chain

After the acquisition of a new *m*-th policy π_*m*_, the robot adds a new Boolean variable *S*_π_*m*__ to the set of state variables, which indicates whether the policy π_*m*_ was used. It takes the value 1 if π was used, and 0 otherwise. To avoid confusion, we also denote the value of the *S*_π_*m*__ with ${\bar{\pi }}_{m}$ when it takes the value 0, and with π_*m*_ when it is 1. Then, the robot continues updating the histograms of the variables as well as calculating the contingency of the experiences, including the new state variable *S*_π_*m*__. Using this method, the robot becomes able to evaluate the contingency of the c-Chains, and as a result, evaluate the contingency related to the new behavior of the caregiver who observed the contingency reproduction of the robot. In previous work (Mahzoon et al., 2016), an example of such a c-Chain was the consistent response of the caregiver to the social referencing behavior of the robot: the robot found that after using the gaze-following skill, if it looks at the caregiver's face, the caregiver will look at the face of the robot as an acknowledgement. Moreover, they showed that in a more complex simulation environment, the robot acquires a longer sequence of actions, up to five sequences.

## 3. Proposed Method

In this section, after discussing the two essential weak points of the previous work (Mahzoon et al., 2016) and our solution for each of them, we describe the mechanism of our proposed method.

### 3.1. Ostensive-Cue Sensitive Learning (OsL)

The first problem of previous work is the synchronization of the teaching phase of the human caregiver with the learning phase of the infant robot. Learning under the non-synchronized environment decreases the learning efficiency of the robot, and causes significant delays in the learning progress. Although distinguishing the teaching phase of the human by the robot seems to be a difficult issue owing to the probable variety of types of teaching in different human subjects, there are several reports in the fields of cognitive science and developmental psychology regarding how human infants treat the synchronization problem and increase the efficiency of learning from adults (see a review Csibra and Gergely, 2011).

Csibra and Gergely addressed the “natural pedagogy” as a human communication system for generic knowledge transmission between individuals (Csibra and Gergely, 2009). They proposed that human infants are “prepared to be at the receptive side of natural pedagogy” and sensitive to learn from the ostensive cues of human adults, such as mutual eye contact between the adults and the infant, or adults' infant-directed speech (motherese). From this statement, we hypothesize that the human adult may inherently or adaptively output the ostensive cues when it tries to teach something to the human infant, or even to the infant robot. Based on this hypothesis, we propose the OsL algorithm for the infant robot as follows: (1) The robot stops moving when it observes an ostensive cue from the human and continues the observation of the human until the signal disappears. This is because the ostensive cue acts as a signal (from our hypothesis) that informs the robot about the human's teaching phase, and notifies the robot to synchronize with it; (2) The robot counts the histogram of the consequent experiences right after the disappearance of the ostensive cue η times (i.e., the learning weight parameter of the OsL algorithm) instead of one time in order to emphasize such experiences. This is because (from our hypothesis) after the ostensive signals, the human would be in the teaching phase and the experiences right after the ostensive cues probably contain more informative concepts compared with other experiences. Using OsL, we expect the robot to increase the efficiency of learning and, as a result, the speed of skill acquisition.

### 3.2. Exclusive Evaluation of Policy (XEP)

The second problem of the previous work is the overestimation of the transition probabilities of the single contingencies, which leads to an underestimation of C˜SJ(e) and/or C˜AJ(e), i.e., Equations (5) and (6), when the robot uses an acquired policy. This leads to the underestimation of the ScC of some experiences, i.e., ${\tilde{C}}_{J}$: Equation (7). The reasons for the overestimation and the underestimation are as follows. Assume that the robot acquired its new *m*-th policy π_*m*_ based on the contingent experience $e_{m}=\left(s_{i}^{t},a_{j}^{t},r_{k}^{t},r_{k}^{t+1}\right)$. Before the robot starts to use π_*m*_, i.e., using the sensorimotor mapping $\left(s_{i}^{t},a_{j}^{t}\right)$, the C˜SJ(e) and C˜AJ(e) of the experience ***e***_*m*_ can be written by the transition probabilities calculated based on the histograms of the variables before acquiring and using π_*m*_, i.e., *p*^*bef*^, as follows:

(8) C˜SJbef(em)=logpbef(rkt+1|sit,ajt,rkt)pbef(rkt+1|sit,rkt),

(9) C˜AJbef(em)=logpbef(rkt+1|sit,ajt,rkt)pbef(rkt+1|ajt,rkt).

However, when the robot starts to use π_*m*_, the probability of taking action $a_{j}^{t}$ in state $s_{i}^{t}$ increases. This fact increases the value of the transition probabilities (1) $p\left(r^{t+1}|s_{i}^{t},r_{k}^{t}\right)$ and (2)$p\left(r^{t+1}|a_{j}^{t},r_{k}^{t}\right)$, i.e., the numerator of the single contingencies: Equations (3) and (4); and the denominator of C˜SJ(e) and C˜AJ(e): Equations (5) and (6). The reasons are (1) for $p\left(r^{t+1}|s_{i}^{t},r_{k}^{t}\right)$: in state $s_{i}^{t}$, the probability of taking action $a_{j}^{t}$ increases owing to the usage of π_*m*_, which is a contingent skill and leads the transition to $r_{k}^{t+1}$ with high probability; and (2) for $p\left(r^{t+1}|s_{i}^{t},r_{k}^{t}\right)$: the probability of having been in state $s_{i}^{t}$ when the action $a_{j}^{t}$ is taken increases owning to the usage of π_*m*_. Assume that the values of the transition probabilities $p\left(r^{t+1}|s_{i}^{t},r_{k}^{t}\right)$ and $p\left(r^{t+1}|a_{j}^{t},r_{k}^{t}\right)$ after the usage of π_*m*_, i.e., denoted by *p*^*aft*^, increase by factors of α and β, respectively, compared to *p*^*bef*^:

(10) $$\begin{array}{l} p^{aft}\left(r_{k}^{t+1}|s_{i}^{t},r_{k}^{t}\right)=\alpha .p^{bef}\left(r_{k}^{t+1}|s_{i}^{t},r_{k}^{t}\right)\;;\;\alpha >1 \end{array}$$

(11) $$\begin{array}{l} p^{aft}\left(r_{k}^{t+1}|a_{j}^{t},r_{k}^{t}\right)=\beta .p^{bef}\left(r_{k}^{t+1}|a_{j}^{t},r_{k}^{t}\right)\;;\;\beta >1 \end{array}$$

Assuming that the value of the transition probability $p\left(r_{k}^{t+1}|s_{i}^{t},a_{j}^{t},r_{k}^{t}\right)$ does not change before and after the usage of π_*m*_ (because the usage of π_*m*_ as a sensorimotor mapping $\left(s_{i}^{t},a_{j}^{t}\right)$ is included in the condition part of the transition probability), the values of C˜SJ(e) and C˜SJ(e) for the experience ***e***_*m*_ after the usage of π_*m*_ can be written as:

(12) C˜SJaft(em)=logpbef(rkt+1|sit,ajt,rkt)α.    pbef(rkt+1|sit,rkt)                       =C˜SJbef(em)−logα                                            ;    α>1,

(13) C˜AJaft(em)=logpbef(rkt+1|sit,ajt,rkt)β.    pbef(rkt+1|ajt,rkt)                       =    C˜AJbef(em)−logβ                                            ;    β>1.

Therefore, ScC of the experience ***e***_*m*_ after the usage of the π_*m*_ will become:

(14) C˜J    aft(em)=min{C˜SJbef(em)−logα,    C˜AJbef(em)−logβ}                         < C˜J    bef(em).

To avoid such an underestimation, we propose to separate the contingency evaluations related to the acquired policies and atomic variables, namely the XEP algorithm. In this algorithm, the system adds an *extra* variable for each sensory and action variable to the system, denoted by ${\hat{S}}_{i}^{t}$ and ${\hat{A}}_{j}^{t}$. When an acquired policy π_*m*_ is used, the system sets the values of ${\hat{S}}_{i}^{t}$ and ${\hat{A}}_{j}^{t}$ to *don't care*. Therefore, the histogram of the values of these variables, denoted by ${ŝ}_{i}^{t}$ and ${â}_{j}^{t}$, are counted only if an acquired policy has not been used. Using the histogram of these variables for the calculation of the transition probabilities of Equations (10) and (11), which are denoted by $\hat{p}$, causes them not to increase even after usage of the policy π_*m*_:

(15) $$\begin{array}{l} {\hat{p}}^{aft}\left(r_{k}^{t+1}|s_{i}^{t},r_{k}^{t}\right)=p^{aft}\left(r_{k}^{t+1}|{ŝ}_{i}^{t},r_{k}^{t}\right)\\ =p^{bef}\left(r_{k}^{t+1}|s_{i}^{t},r_{k}^{t}\right), \end{array}$$

(16) $$\begin{array}{l} {\hat{p}}^{aft}\left(r_{k}^{t+1}|a_{j}^{t},r_{k}^{t}\right)=p^{aft}\left(r_{k}^{t+1}|{â}_{j}^{t},r_{k}^{t}\right)\\ =p^{bef}\left(r_{k}^{t+1}|a_{j}^{t},r_{k}^{t}\right). \end{array}$$

Therefore, when the XEP algorithm is used, the value of C˜SJ and C˜AJ for the experience ***e***_*m*_, which are denoted by C^SJ and C^AJ, after the usage of π_*m*_ will be:

(17) C^SJaft(em)=logpbef(rkt+1|sit,ajt,rkt)p^aft(rkt+1|sit,rkt)                      =    C^SJbef(em),

(18) C^AJaft(em)=logpbef(rkt+1|sit,ajt,rkt)p^    aft(rkt+1|ajt,rkt)                      =C^AJbef(em).

As the result, the ScC of the experience ***e***_*m*_ when the XEP algorithm is used, which is denoted by ${\hat{C}}_{J}$, after the usage of π_*m*_ will be:

(19) C^J    aft(em)=min{C^SJaft(em),    C^AJaft(em)}                     =C^J    bef(em).

With respect to Equation (19) and Inequation (14), it can be concluded that the XEP algorithm is able to solve the underestimation problem of the previous work (Mahzoon et al., 2016), and is expected to increase the accuracy of the contingency evaluation^1^.

### 3.3. Mechanism

**Figure 2:**
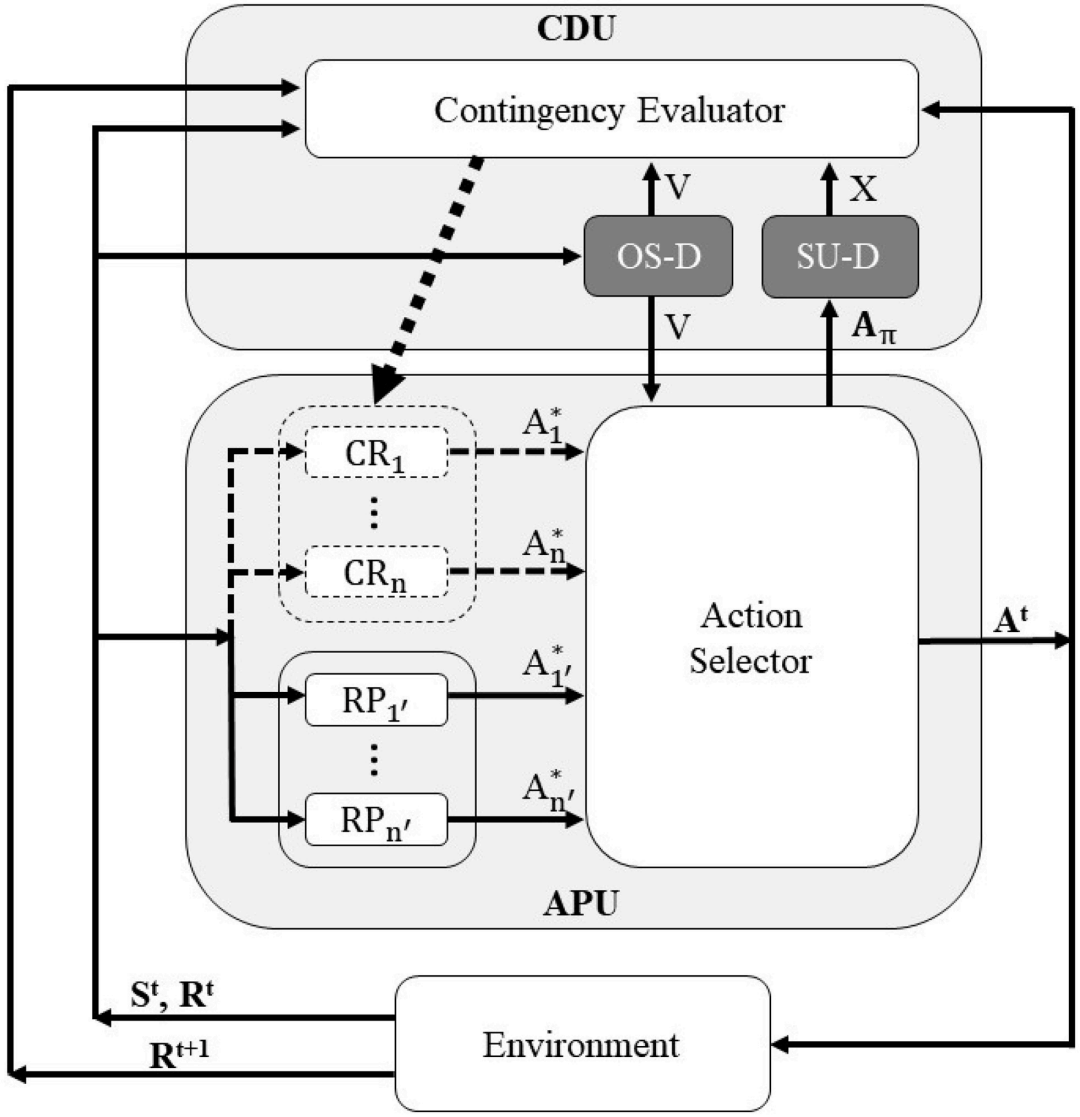
System schema of the proposed mechanism, consisting of two main parts: Contingency Detection Unit (CDU) and Action Producing Unit (APU). Contingency Reproducer (CR), Reaction Producer (RP), and Action Selector compose the APU, while the Contingency Evaluator, Ostensive Signal Detector (OS-D) and Skill Usage Detector (SU-D) form the CDU. The new components of the proposed mechanism are shown with the darker color (OS-D and SU-D). *V*, *X*, and *A*_π_ indicates the controlling signals described in section 3.3.1. In each time step, the robot outputs the action *A*^*t*^ based on its current states *S*^*t*^ and *R*^*t*^, and observes the resultant transition of the environment, i.e., *R*^*t*+1^.

Figure 2 shows the schema of the proposed system. It consists of two main parts: the Contingency Detection Unit (CDU) and the Action Producing Unit (APU). The APU is responsible for determining the output action in each time step, while the CDU evaluates the contingency of the experiences. At each time step *t*, the robot observes the environment and stores the results of the current observation in ***S***^*t*^ and ***R***^*t*^ (bottom part of the figure). They are sent to the APU, and the APU decides about the outputting action for each joint of the robot ***A***^*t*^, based on the input data ***S***^*t*^ and ***R***^*t*^. After taking the action, the robot again observes the environment, and stores the resultant observation in the resultant variable ***R***^*t*+1^ (bottom part of the figure). Simultaneously, in each time step, the CDU gets the values of all of the variables, and evaluates the contingency of the experiences. If the CDU detects an experience as a contingent one, it adds a new Contingency Reproducer (CR in Figure 2) to the APU, which enables the APU to reproduce the found contingency. In the remainder of this section, each component of the CDU and APU are explained in detail.

#### 3.3.1. Contingency Detection Unit (CDU)

In each time step, the CDU (1) evaluates the contingency of the experiences, and (2) if a contingent experience is detected, it adds a new CR to the APU, which enables the robot to reproduce the found contingency. The CDU consists of three components: the Contingency Evaluator, Ostensive Signal Detector (OS-D), and the Skill Usage Detector (SU-D).

##### 3.3.1.1. Contingency Evaluator

This unit calculates the contingencies of the experiences based on the histograms of the experiences, using the method described in section 2.2. If the experience $e=\left(s_{i}^{t},a_{j}^{t},r_{k}^{t},r_{k}^{t+1}\right)$ is distinguished as a contingent one, it adds a new CR to the APU, which contains the values of the variables of the found contingent experience ***e***, i.e., $s_{i}^{t},a_{j}^{t},r_{k}^{t}$ and $r_{k}^{t+1}$. After that, the Contingency Evaluator continues the evaluation of the contingencies, including the c-Chains (see section 2.3), as well as the process of adding further CRs to the system.

##### 3.3.1.2. OS-D

The OS-D gets the current state of the robot ($S_{i}^{t}$ and $R_{k}^{t}$). If it detects that these variables include an ostensive cue from the human, it sends the *stop signal V* to the Contingency Evaluator as well as the Action Selector. This signal causes the Contingency Evaluator to pause counting the histograms, and the Action Selector to make the robot to keep looking at the human and stop its movement. Additionally, it sends the learning weight parameter η (see section 3.1) to the Contingency Evaluator. When the ostensive cue disappears, the stop signal *V* is canceled simultaneously, which makes the Contingency Evaluator and Action Selector restart their functions. In this paper, mutual eye contact with the human caregiver is implemented as the only ostensive cue of the interaction.

##### 3.3.1.3. SU-D

The SU-D gets the information regarding the usage of the policies in each time step from the Action Selector, and informs the Contingency Evaluator if any policy has been used at the current moment. To this end, the SU-D gets the values of the Boolean variable *A*_π_*m*__ from the the Action Selector, which indicates if the *m*-th policy is currently used, and sends the Boolean signal *X* to the Contingency Evaluator, which is calculated as follows:

(20) $$\begin{array}{l} X=\overset{N_{\pi }}{\underset{m=1}{\vee }}A_{\pi_{m}}\;, \end{array}$$

where *N*_π_ denotes the number of the policies that the robot has acquired until now. If the value of the signal *X* is true, the Contingency Evaluator sets the value of the extra variables ${\hat{S}}_{i}^{t}$ and ${\hat{A}}_{j}^{t}$ to *don't care*, as described in section 3.2.

#### 3.3.2. Action Producing Unit (APU)

As shown in Figure 2, the APU is equipped with three components, the Reaction Producers (RP), Contingency Reproducers (CR), and Action Selector. At the beginning of the interaction, the APU contains no CRs and selects the actions of the robot at each time steps from the suggested actions of the RPs, denoted by $A_{1^{\prime }}^{*}$ to $A_{n^{\prime }}^{*}$ in Figure 2 where *n*′ indicates the number of RPs in the system. Continuing the interaction with the caregiver leads the CDU to find contingent experiences and add CRs to the APU, which include specific sensorimotor mappings, as described in section 3.3.1. Similar to the RPs, the CRs send their suggested actions to the Action Selector, denoted by $A_{1}^{*}$ to $A_{n}^{*}$ in Figure 2, where *n* indicates the number of CRs acquired by the robot. Therefore, after adding CRs to the system, the Action Selector needs to choose the outputting action command to each joint of the robot from all of the candidates: $A_{m}\in \left\{A_{1}^{*},A_{2}^{*},\cdots \;,A_{n}^{*},A_{1^{\prime }}^{*},A_{2^{\prime }}^{*},\cdots \;,A_{n^{\prime }}^{*}\right\}$ where *m* indicates the *m*-th joint of the robot.

##### 3.3.2.1. Contingency Reproducer (CR)

The CR gets the current state of the robot and outputs its suggested action to the Action Selector, based on its sensorimotor mapping. Additionally, it sends the reliability *Z* to the Action Selector, which indicates the certainty of the transition to the expected state if the Action Selector selects its suggested action as the output action of the robot. Assume the *m*-th CR was added to the system based on the contingent experience $e_{m}=\left(s_{i}^{t},a_{j}^{t},r_{k}^{t},r_{k}^{t+1}\right)$. If the current state $S_{i}^{t}$ and $R_{k}^{t}$ are the same as $s_{i}^{t}$ and $r_{k}^{t}$ of the CR, it outputs the candidate action $a_{j}^{t}$ to the Action Selector. Otherwise, it does not send any candidate. In this paper, the CR sends the ScC of the experience ***e***_*m*_, i.e., ${\hat{C}}_{J}\left(e_{m}\right)$, as its reliability *Z*_*m*_ to the Action Selector.

##### 3.3.2.2. Reaction Producer (RP)

The RP gets the current state of the robot and outputs a pre-programmed reaction, which is sent to the Action Selector as the suggested action of the RP. Also it sends a constant value α_*m*_ as its reliability *Z*_*m*_ to the Action Selector, where *m* indicates the *m*-th RP. For the sake of simplicity, in this paper we considered only one RP for the system, which outputs a random action for any input state.

##### 3.3.2.3. Action Selector

The Action Selector chooses the output action for each joint of the robot at each time step. A soft-max action selection was utilized to choose the output from the candidates. Assume that for the *j*-th joint of the robot, the number of RPs and CRs which send the candidate action to the Action Selector, namely inputting components, are $N_{j}^{R}$ and $N_{j}^{C}$, respectively. At each time step, the probability of selecting the suggested action of the inputting component *i* for the joint *j* is calculated based on their reliability as follows:

(21) $$\begin{array}{l} P_{i}^{j}=\frac{\exp\left(Z_{i}/\tau \right)}{\sum_{k\in N_{j}^{R}+N_{j}^{C}}\exp\left(Z_{k}/\tau \right)}, \end{array}$$

where *Z*_*i*_ indicates the reliability of the inputting component *i*, and τ is a temperature constant. Note that if *Z*_*i*_ is less than the omission threshold *C*_*O*_, the Action Selector does not consider the inputting component *i* in Equation (21) and $P_{i}^{j}$ for that component is set to zero. This mechanism enables the robot to have a chance to omit any acquired skill, which might be acquired owing to the noise, lack of sufficient experiences, or other error factors. We set *C*_*O*_ = *C*_*T*_−ε, where the *C*_*T*_ is the skill acquisition threshold (see section 2.2), and ε is a constant value. Additionally, when more than two CRs with the same suggested action and different c-Chain length exist in the inputting components, the Action Selector considers only the one with the longer c-Chain length as the inputting component, and ignores the others, i.e., sets their $P_{i}^{j}$ values to zero.

When the suggested output of the *m*-th CR with the policy π_*m*_ is selected as the output, the Action Selector sets the value of the Boolean variable *A*_π_*m*__ to 1. It sends *A*_π_*m*__ to the SU-D in each time step to inform the SU-D about the usage of the skills. Also, when the Action Selector gets the stop signal *V* from the OS-D, it stops outputting new action commands to the joints of the robot until the stop signal disappears.

## 4. Experiment and Result

In this section, the results of the real-world robot experiment with human subjects are reported. To evaluate the effect of the proposed methods, i.e., the XEP and OsL algorithms, the performances of four different learning mechanisms are compared, of which the CDU consists of (1) neither the SU-D nor the OS-D, (2) only the SU-D, (3) only the OS-D, and (4) both the SU-D and the OS-D. In the remainder of this paper, they are referred to as the previous method, XEP method, OsL method, and proposed method, respectively. This study was carried out in accordance with the recommendations of the ethics committee for research involving human subjects at the Graduate School of Engineering Science, Osaka University. The protocol was approved by the ethics committee for research involving human subjects at the Graduate School of Engineering Science, Osaka University. All subjects gave written informed consent in accordance with the Declaration of Helsinki.

### 4.1. Subjects, Apparatus, and Procedure

**Figure 3:**
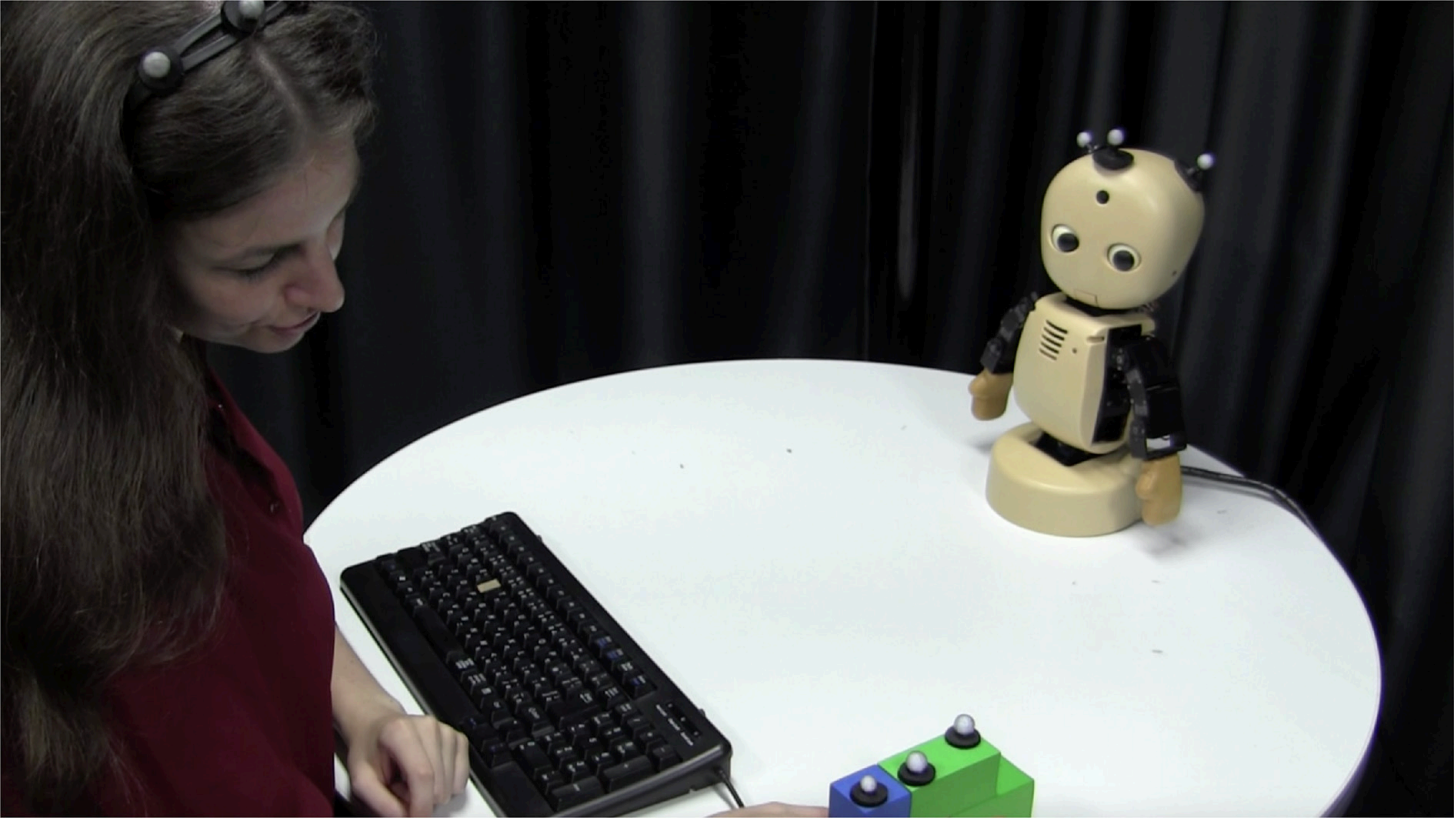
The environment of the subject experiment. The subjects were asked to teach the current position of the toy to the robot. Also, they were asked to push a button of the keyboard to express that they are smiling and praising the robot at the moment. The consent for publication of this image was obtained from the participant of this image by using a written informed consent.

Figure 3 shows the environment of the experiment, which was designed based on the concepts explained in section 2.1 and Figure 1. The human subject was asked to sit opposite the humanoid infant robot and interact with it naturally, as when he/she interacts with a human infant. The subject was asked to play with the robot using a toy on the table and draw the attention of the robot to the toy by teaching the current position of the toy as well as the name, color, shape, or other features of it. It is explained to the subject that the robot may learn some social skills from the behavior of the subject, and start to use them. When the robot uses a learned skill, the LEDs on the face of the robot turn on temporarily. The subject was asked to praise the robot by hitting a specific key on the keyboard when the robot finds the toy by using an acquired skill, i.e., when the LEDs turn on. Additionally, he/she was asked to change the position of the toy around every 20 s. The experiment was conducted for 800 time steps, i.e., around 40–50 min of interaction. After every 200 steps, i.e., around 10 min, the experiment was paused and the subject was asked to answer a simple questionnaire about the interaction, which may take <2 min (see section 4.5).

Twelve sessions were conducted for each four conditions described in section 4 using different human subjects, i.e., totally 48 adults: 30 males and 18 females. Before the main experiment, a test phase of 2 min was conducted to make everything clear for the subject. In this experiment, each time step was set to 2–2.5 s based on the complexity of the robot's internal calculations. Additionally, when the robot used a complex skill, the LEDs were set to temporally *flash* with frequency of *f* = 2*Hz* instead of just turning on; but the subject was not told about it.

### 4.2. Variables and Parameters

In this experiment, the number of objects was set to 1, and the position of the object on the table was quantized to 3 regions: left side, right side, and the middle of the table. Based on our experience, the other parameters were set as follows: for the CDU, (*C*_*T*_, θ, η) = (0.7, 5, 2), and for the APU, (α_*m*_, τ, ε) = (0, 0.4, 0.1).

Table 1 shows the initial variables used in this experiment. For the perception ***S***, two variables were prepared: the gaze direction of the caregiver (*S*_1_) and the observation of the object (*S*_2_). *S*_1_ takes the values *f*_1_, *f*_2_, and *f*_3_ when the robot recognizes that the caregiver is looking at the left, right, and the middle of the table, respectively. It takes the value *f*_*r*_ when the robot detects that the caregiver is looking at it, and the value *f*_ϕ_ when the robot cannot detect the direction of the gaze of the caregiver. *S*_2_ takes the value *o* when the robot detects the object, and *o*_ϕ_ when no object is detected. A motion capture system was utilized to detect the gaze direction of the caregiver as well as the position of the object in each time step.

**Table 1:** Variables of the robot for the experiment.

**Type**	**Variable name**	**Symbol**	**Elements**
***S***	Caregiver's gaze direction	*C*	*S*_1_ = {*f*_1_, *f*_2_, *f*_3_, *f*_*r*_, *f*_ϕ_}
	Object	*O*_*s*_	*S*_2_ = {*o, o*_ϕ_}
***A***	Gaze shifting	*G*	*A*_1_ = {*g*_1_, *g*_2_, *g*_3_, *g*_*c*_}
	Hand Gesture	*H*	*A*_2_ = {*h*_1_, *h*_2_, *h*_3_, *h*_4_}
***R***	Frontal face of caregiver	*F*	$R_{1}=\left\{{\bar{r}}_{1},r_{1}\right\}$
	Profile of caregiver	*P*	$R_{2}=\left\{{\bar{r}}_{2},r_{2}\right\}$
	Object	*O*_*r*_	$R_{3}=\left\{{\bar{r}}_{3},r_{3}\right\}$
	Praise from caregiver	*W*	$R_{4}=\left\{{\bar{r}}_{4},r_{4}\right\}$

For the actions of the robot ***A***, two variables were prepared: gaze shift (*A*_1_) and the hand gesture of the robot (*A*_2_). *A*_1_ takes the values *g*_1_, *g*_2_, and *g*_3_ when the robot shifts its gaze and looks at the left, right, and the middle of the table, respectively. It takes the value *g*_*c*_ when the robot looks at the caregiver's face. *A*_2_ takes the values *h*_1_, *h*_2_, *h*_3_, and *h*_4_, which indicate the different types of hand gestures known by the robot. In this experiment, each values of the *h*_*j*_ were implemented as a different degree of the pitch of the robot's arm.

For the resultant perception ***R***, four Boolean variables were considered: the frontal face of the caregiver (*R*_1_), the profile (face) of the caregiver (*R*_2_), the observation of the object (*R*_3_), and the praise from the caregiver(*R*_4_). They take the value 1 if the frontal face, the face in profile, the object and the smile of the caregiver are observed by the robot. Otherwise, they take the value 0. To avoid confusion, the values of *R*_1_, *R*_2_, *R*_3_, and *R*_4_ are also denoted with *r*_1_, *r*_2_, *r*_3_, and *r*_4_ when they take 1, and with ${\bar{r}}_{1}$, ${\bar{r}}_{2}$, ${\bar{r}}_{3}$, and ${\bar{r}}_{4}$ when they are 0, respectively. In the experiment, to detect the values of *R*_1_, *R*_2_, and *R*_3_, the motion capture system was utilized, while the praise from the caregiver, i.e., *R*_4_, was expressed by the caregiver hitting a specific key on the keyboard. Also, to avoid confusion of the variables and to facilitate further discussions, each variable is mentioned with the symbol indicated in Table 1 in the remainder of the paper.

### 4.3. Developmental Process of Social Skill Acquisition

**Figure 4:**
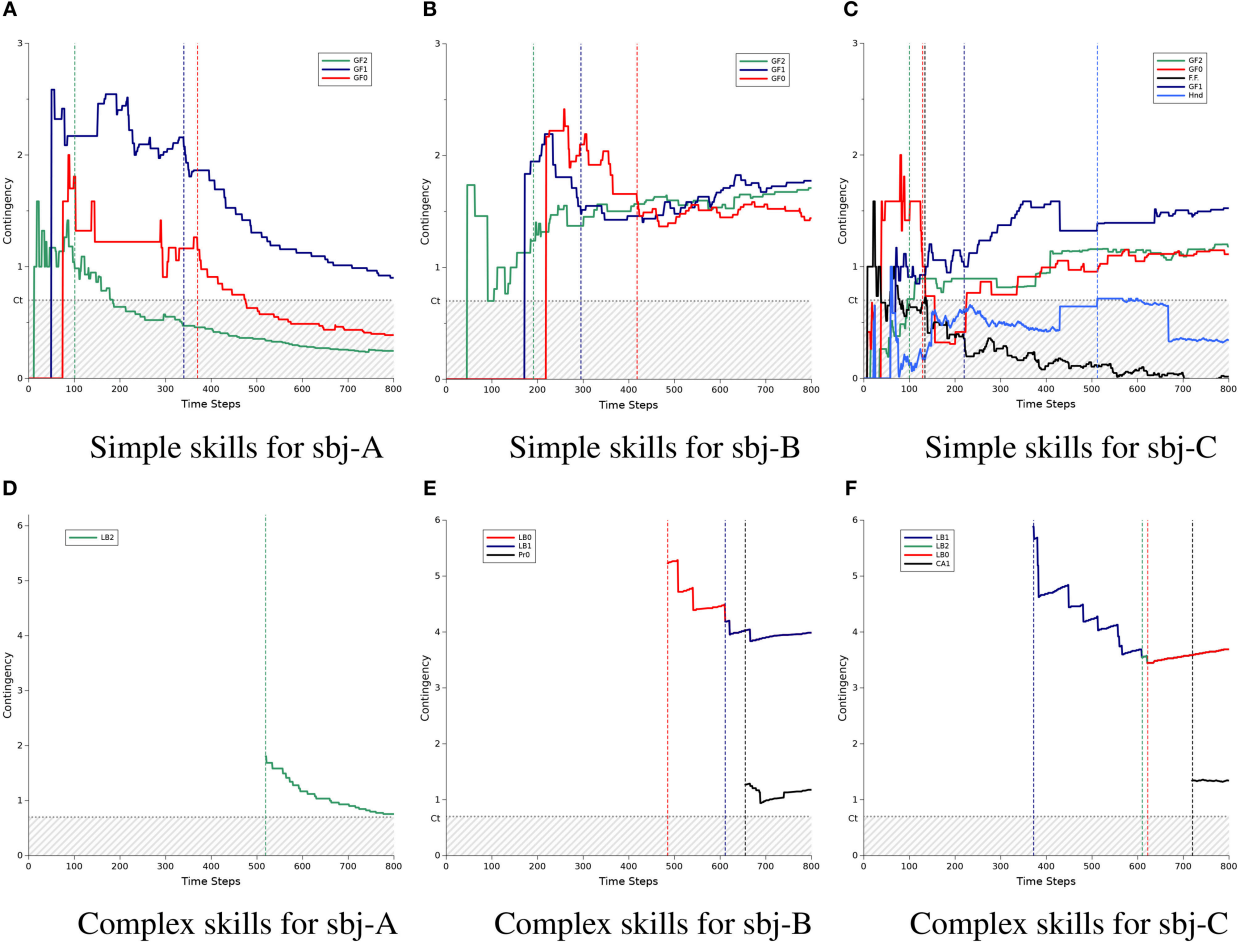
Developmental process of the acquisition of social skills by the robot: a comparison among three participants (sbj-A, sbj-B, and sbj-C). For each subject, the process was shown for simple skills (top three graphs) and complex skills (bottom three graphs). The horizontal axes indicate the time step of the experiment (ends at 800, which is equal to around 40 min.), while the vertical axes labeled contingency indicate the amount of the calculated contingency using equation (7). Each sub-figure include the time courses of contingency for some sample set of experience ***e***, which are mentioned with a name such as Gaze-Following or Looking-Back in the legend of the figures. *C*_*t*_ indicated the threshold for the contingency of ***e*** to be acquired as a skill. The vertical dotted lines indicate the timing of the acquisition of a ***e*** as a skill, where its color represents which experience is acquired. Note that 2 lines (red and blue) in **(E)** and 3 lines (blue, green, and red) in **(F)** are overlapped, but represent contingency for different experiences. **(A)** Simple skills for sbj-A. **(B)** Simple skills for sbj-B. **(C)** Simple skills for sbj-C. **(D)** Complex skills for sbj-A. **(E)** Complex skills for sbj-B. **(F)** Complex skills for sbj-C.

Before the statistical comparison of performance of the different methods, we first show the developmental process of social skill acquisition by the robot using some examples from the experimental results of three subjects. Tables 2–**4** show the acquired skills by the robot during the experiment with these subjects, namely sbj-A, sbj-B, and sbj-C, respectively. While the robot utilized the previous method in the case of sbj-A, it used the proposed method for the case of sbj-B and sbj-C. Additionally, Figure 4 shows the time course of the evaluated amount of contingencies related to each acquired skills indicated in Tables 2–**4**.

**Table 2:** Acquired social skills by the robot for the sbj-A.

**ID**	**Step**	**Level**	**Label**	***r*^*t*^**	***s*^*t*^**	***a*^*t*^**	***r*^*t*+^**	**Interpreted function**
π_1_	101	1	**Gaze-Following-2**	${\bar{r}}_{3}$	*f*_2_	*g*_2_	*r*_3_	Gaze Following (middle)
π_2_	340	1	**Gaze-Following-1**	${\bar{r}}_{3}$	*f*_1_	*g*_1_	*r*_3_	Gaze Following (right)
π_3_	370	1	**Gaze-Following-0**	${\bar{r}}_{3}$	*f*_0_	*g*_0_	*r*_3_	Gaze Following (left)
π_4_	519	2	**Looking-Back-2**	${\bar{r}}_{4}$	π_1_	*g*_*c*_	*r*_4_	Looking Back (after Gaze-Following-2)

In these tables, the “ID” column indicates the ID of the contingency reproducer (CR),“Step” indicates the time-step at which that the CR was acquired, “Level” indicates the length of the c-Chain of the acquired CR, “Label” shows the symbol of the CR which may be used to refer to it by the subsequent CRs (and also it is used in Figure 4 to indicate each CR), the column of “*r*^*t*^, *s*^*t*^, *a*^*t*^, and *r*^*t*+1^” indicate the experience ***e*** on which the CR was created, and finally, the interpretation of the CR is given based on the behavior of the robot when it uses the CR in the column of “Interpreted Function.”

In Figure 4, the graphs of the simple and complex skills are separated: the top part (Figures 4A–C) for the simple skills and the bottom part (Figures 4D–F) for the complex ones. Each column of the figure indicates the result of each subject: from the left to right for sbj-A, sbj-B, and sbj-C, respectively. In each graph, the threshold of the contingency acquisition *C*_*T*_ is shown with the horizontal dotted gray line, and the hatched area indicates the values less than the threshold; while the vertical dashed lines indicate the time-step that each CR was acquired (the color is the same as that of the corresponding CR indicated in the legend of the graphs). Note that the order of the CRs at the legend of the graphs are the same as the order in which they were acquired. Also, the colors of the lines for Gaze-Following and Looking-Back are set based on their corresponding directions: red, blue, and green for the left, right, and the middle of the table, respectively.

According to the first row of Table 2, in the case of the sbj-A, where the robot was using the previous method, the robot acquired its first CR π_1_ at *t* = 101, which for the inputs $\left({\bar{r}}_{3},f_{2}\right)$, outputs the action *g*_2_ to observe *r*_3_. In other words, this CR indicates that when the robot recognizes that the human subject is looking at the middle of the table (*f*_2_), if the robot shifts its gaze to the same position, i.e., the middle of the table (*g*_2_), then the robot can find the object (transition of ${\bar{r}}_{3}$ to *r*_3_). Using this CR, the robot can produce the gaze following behavior (to the middle of the table). It is noted by the symbol **Gaze-Following-2** (where the number indicates the position of the table) and the time course of the calculated contingency of the experience related to **Gaze-Following-2**, i.e., $e_{\text{GF}2}=\left(f_{2},g_{2},{\bar{r}}_{3},r_{3}\right)$, is shown in Figure 4A with the green line. From the beginning of the interaction, the contingency of **Gaze-Following-2** goes higher than the threshold *C*_*T*_ (the vertical dashed line), and after a while [namely, after experiencing the ***e***_GF2_ more than θ (=5) times], it is acquired as the first CR of the robot. The vertical green dashed line around *t* = 100 in Figure 4A shows the timing of the acquisition of this CR, which corresponds to the value of “Step" in π_1_, Table 2. As shown in the figure, the value of the contingency of **Gaze-Following-2** was 0.98 at the acquisition time, while it decreases to 0.25 at the end of the experiment.

Following the time courses of the other contingencies in Figure 4A we can see that the robot acquired gaze-following skill to the right and left side of the table at *t* = 340 and *t* = 370, respectively (blue and red lines, corresponding with π_2_ and π_3_ of Table 2, respectively). After the acquisition of the skills, the robot starts to use them as described in section 3.3.2.3. At *t* = 519, the robot found a contingent relationship between using **Gaze-Following-2** and being praised by the human, and acquired new CR with a level of 2 (the green line in Figure 4D and π_4_ in Table 2). This CR tells the robot that after using the gaze following to the middle of the table ($s^{t}=\pi_{1}$), if it shifts gaze to the human ($a^{t}=g_{c}$), then the robot would be praised by the human (transition of $r^{t}={\bar{r}}_{4}$ to $r^{t+1}=r_{4}$). In this paper, we refer to this behavior as looking back behavior (**Looking-Back**). Acquisition of the **Looking-Back-2** would be due to the specific praising behavior of the human during the experiment (see section 4.1). This CR shows that the robot develops the acquired skills (such as **Gaze-Following-2**) to more complex ones (such as **Looking-Back-2**), which enables the robot to have longer interaction sequence with the human subject.

However, in the case of the sbj-A, the implemented method was the previous method. As described in section 3.2, the previous method has no mechanism to prevent the underestimation of contingencies after the acquisition of the CRs. Therefore, in Figures 4A,D, the contingency of the acquired CRs decreased after the acquisition of each CRs. As result, the contingency of the **Gaze-Following-2** and **Gaze-Following-0** (green and red lines) become less than the omission threshold *C*_*O*_ (=0.6), i.e., 0.1 lower than the threshold *C*_*T*_ in the graphs, and the Action Selector would stop using them. Additionally, a smaller value of the contingencies reduces the value of *Z*, which leads the Action Selector to use the CRs with less probability (see Equation 21). Therefore, in the previous method, although the robot could acquire simple and complex skills, it may not be able to use them properly.

Table 3, Figures 4B,E show the result of the experiment of sbj-B, in which the proposed method was implemented on the robot. Compared with the case of the sbj-A (which the previous method was implemented), the contingency of the **Gaze-Following**s do not decrease to less than (or close to) the omission threshold and, as a result, the robot could acquire more complex skills (two **Looking-Back**s and one **Looking-Profile**). Considering the probable irregular behavior of the human against the robot or the noise of the environment in the real-world interaction, preventing the underestimation of the contingencies seems to be very important, as shown in this example. Note that if the subjects had praised the robot when the robot found the object by using the Gaze-Following skill with high probability, the value of the contingency of **Looking-Back** is theoretically 4 with respect to Equation (7); assuming that the numerator of Equations (5) and (6) are approximately 1 due to the accurate praising behavior of the caregiver, while the denominator of Equation (5) is 0.25 because if the robot chooses the gaze action *g*_*c*_ from the four possible ones *g*_1_,*g*_2_,*g*_3_, and *g*_*c*_ it would be praised, and the denominator of Equation (6) is at most 0.25 because it is equal to the probability that the robot had found the object before the robot takes the action *g*_*c*_. During the experiment, although both the sbj-A and sbj-B seemed to praised the robot with same manner, the contingency of the **Looking-Back-2** (green line in Figure 4D) for the sbj-A became 0.76 at the end of the experiment, while in the case of the sbj-B, it became 3.99 for both **Looking-Back-0** and **Looking-Back-1** (red and blue lines in Figure 4E), which is very close to the value of the theoretical calculation. Note that the overlap of the **Looking-Back**s is due to the small number of the experiences related to the **Looking-Back**s, which makes the transition probabilities of their contingency evaluation very close to each other.

**Table 3:** Acquired social skills by the robot for sbj-B.

**ID**	**Step**	**Level**	**Label**	***r*^*t*^**	***s*^*t*^**	***a*^*t*^**	***r*^*t*+1^**	**Interpreted Function**
π_1_	191	1	**Gaze-Following-2**	${\bar{r}}_{3}$	*f*_2_	*g*_2_	*r*_3_	Gaze Following (middle)
π_2_	295	1	**Gaze-Following-1**	${\bar{r}}_{3}$	*f*_1_	*g*_1_	*r*_3_	Gaze Following (right)
π_3_	418	1	**Gaze-Following-0**	${\bar{r}}_{3}$	*f*_0_	*g*_0_	*r*_3_	Gaze Following (left)
π_4_	485	2	**Looking-Back-0**	${\bar{r}}_{4}$	π_3_	*g*_*c*_	*r*_4_	Looking Back (after Gaze-Following-0)
π_5_	611	2	**Looking-Back-1**	${\bar{r}}_{4}$	π_2_	*g*_*c*_	*r*_4_	Looking Back (after Gaze-Following-1)
π_6_	655	2	**Looking-Profile-0**	${\bar{r}}_{2}$	π_3_	*g*_*c*_	*r*_2_	Finding Profile (after Gaze-Following-0)

Following the time courses of Figure 4E, finally a new complex skill **Looking-Profile-0** is acquired. This CR (see π_6_ of Table 3) causes the robot to look at the human (*g*_*c*_) after following its gaze (π_3_) to find human's face in profile (transition of ${\bar{r}}_{2}$ to *r*_2_). This skill was specific to the sbj-B; it seems that he tended to show his face in profile to the robot when the robot succeeded to find the object by using the **Gaze-Following** skills, probably because he was concentrating to push the correct button of the keyboard to praise the robot while the keyboard was on the right side of the table in the case of the sbj-B. The acquisition of this kind of subject-specific skills shows that the proposed mechanism has the potential of evaluating various kind of human behaviors based on the different interaction manner of the subjects.

Figures 4C,F show the result of another subject, i.e., sbj-C, which the robot was implemented with the proposed method. The result shows more complex and interesting process of the contingency evaluation, acquisition, and omission by the robot. The details of the acquired skills are listed in Table 4. After acquiring the gaze-following skill to the middle and the left side of the table (**Gaze-Following-2** and **Gaze-Following-0**, the green and red lines in Figure 4C), the robot acquired a skill named **Frontal-Face** (the black line), which makes the robot to look at the human (*g*_*c*_) to find his/her frontal face (*r*_1_), when no object was detected (*o*_ϕ_) at *t* = 134 (see π_3_ in Table 4). However, finding the frontal face of the human is due to the single effect of the action *g*_*c*_, but not the joint effect of the state *o*_ϕ_ and action *g*_*c*_ (see section 2.2 for the details of the single and joint effects). Therefore, as shown in the figure, the contingency of the **Frontal-Face** was reduced to less than the omission threshold and as a result, the **Frontal-Face** would not be selected by the Action Selector anymore. The acquisition and omission of this CR shows an example of how the proposed mechanism may acquire a non-contingent skill, use it, update the consequent of the usage of the skill, and finally recognize it as a non-contingent one and stop using it.

**Table 4:** Acquired social skills by the robot for the sbj-C.

**ID**	**Step**	**Level**	**Label**	***r*^*t*^**	***s*^*t*^**	***a*^*t*^**	***r*^*t*+1^**	**Interpreted Function**
π_1_	100	1	**Gaze-Following-2**	${\bar{r}}_{3}$	*f*_2_	*g*_2_	*r*_3_	Gaze Following (middle)
π_2_	129	1	**Gaze-Following-0**	${\bar{r}}_{3}$	*f*_0_	*g*_0_	*r*_3_	Gaze Following (left)
π_3_	134	1	**Frontal-Face**	${\bar{r}}_{1}$	*o*_ϕ_	*g*_*c*_	*r*_1_	Finding Frontal Face
π_4_	220	1	**Gaze-Following-1**	${\bar{r}}_{3}$	*f*_1_	*g*_1_	*r*_3_	Gaze Following (right)
π_5_	372	2	**Looking-Back-1**	${\bar{r}}_{4}$	π_4_	*g*_*c*_	*r*_4_	Looking Back (after Gaze-Following-1)
π_6_	512	1	**Hand-Motion**	${\bar{r}}_{3}$	*f*_1_	*h*_2_	*r*_3_	Finding Object by Hand
π_7_	610	2	**Looking-Back-2**	${\bar{r}}_{4}$	π_1_	*g*_*c*_	*r*_4_	Looking Back (after Gaze-Following-2)
π_8_	622	2	**Looking-Back-0**	${\bar{r}}_{4}$	π_2_	*g*_*c*_	*r*_4_	Looking Back (after Gaze-Following-0)
π_9_	720	3	**Check-Again-1**	${\bar{r}}_{3}$	π_5_	*g*_1_	*r*_3_	Check Again the Object

After the **Frontal-Face**, the robot acquired **Gaze-Following-1**, developed it to **Looking-Back-1**, and acquired another non-contingent skill named **Hand-Motion**, which indicates that the robot can find the object by hand gesture *h*_2_. Since there seemed to be no relation between finding the object and the hand gestures of the robot, therefore the contingency of the **Hand-Motion** was reduced to less than the omission threshold after a while. Then, the robot acquired **Looking-Back-2** and **Looking-Back-0**, and finally acquired another complex skill with the level of 3, named “Check Again”: **Check-Again-1**. This CR informs the robot after using **Looking-Back-1** (π_5_), if it looks at the right side of the table (*g*_1_), it can find the object again (*r*_3_). In other words, when the robot detects that the human is looking at the right side of the table, it follows the gaze of the human and looks at the right side using **Gaze-Following-1** to find the object (π_4_ in Table 4), then looks back at the human using **Looking-Back-1** to be praised (π_5_ in the table), and then, looks at the right side again using **Check-Again-1** to see the object, again (π_9_ in the table).

To summarize this section, we compared a result of one of the best cases of the previous method (sbj-A) with two cases from our proposed method: the case of sbj-B, in which the robot had a moderate performance and the case of sbj-C, in which the robot had a higher performance. In the cases of sbj-B and sbj-C, the robot was able to prevent the underestimation of the contingencies which occurred after the acquisition of the CRs in the previous method. This underestimation can be seen in the case of sbj-A. As a result, the robot could acquire more complex skills in these cases. This was due to the contribution of the XEP algorithm. Moreover, the averages of the time steps spent for the acquisition of simple and complex skills were smaller in these cases. This was due to the contribution of the OsL algorithm. The faster skill acquisition also resulted in the acquisition of more complex skills, concerning the limitation of the time in the real-world experiment.

### 4.4. Quantitative Analysis of Performance

In this section, the effect of the proposed algorithms on the performance of the system was explored. As the measure of the performance analysis, (1) the coverage of Gaze-Following, (2) the coverage of Looking-Back, (3) the time required to learn Gaze-Following, (4) the time required to learn Looking-Back, (5) the number of the acquired non-contingent skills, and (6) the number of the expected transition, was elected and the mean of each performance measure was compared among the experiment conditions. For each performance measure, a 2 × 2 ANOVA was conducted with two between subject factors OsL (0 or 1) and XEP (0 or 1), where 1 indicates that the algorithm was adopted and 0 indicates it was not. Also, a *post-hoc* power analysis was conducted to determine the observed power (1−β) of the test, computed using α = 0.05. In the following three sections, the definition of each performance measure, the result of the statistical tests, and the discussion about the result was proposed, respectively.

#### 4.4.1. Performance Measure

For (1) the coverage of Gaze-Following and (2) the coverage of Looking-Back, the coverage of the acquired Gaze-Following and Looking-Back were calculated in terms of percentage, respectively, where 100% means that the robot learned the skill related to all positions: left, right, and middle of the table. With respect to the instructions of the experiment, the subjects would try to draw the attention of the robot to the object; therefore, the contingency of the Gaze-Following is expected to exist in the interaction, and had to be learned by the robot. Moreover, praising process of the caregiver would lead to the existence of the contingency of Looking-Back in the interaction and had to be learned by the robot, as well. Therefore, the coverage of Gaze-Following and Looking-Back seems be fair and adequate for comparing the learning performance of the systems; for the simple and the complex skills, respectively.

For (3) the time required to learn Gaze-Following and (4) the time required to learn Looking-Back, the average time steps required for learning Gaze-Following and Looking-Back for all three positions, i.e., left, right, and middle of the table; was considered, respectively. If a skill was not acquired, the value was set to 800, i.e., the total time of the experiment. These measures show the learning speed of the system, specifically the learning of the simple and complex skills, respectively.

On the other hand, the OsL uses weighted learning, which may increase the acquisition of the non-contingent skills; and the XEP may compensate it by increasing the accuracy of the contingency evaluation. For that, (5) the number of the acquired non-contingent skills, was considered to be compared among the conditions. These skills were defined as the ones apart from Gaze-Following, Looking-Back, Looking-Profile, and Check-Again. This measure is expected to reflect the non-efficiency of the learning mechanism of the robot.

Finally, the predictability of the learned skills was compared to evaluate the usability of the acquired skills of the robot. It was denoted as (6) the number of the expected transition; and calculated by the average number of the successful expected transitions of the environment conducted by utilizing the learned behaviors. For instance, if the Gaze-Following was used and as a result the robot could find the object, it was counted as a successful expected transition.

#### 4.4.2. Result of Comparison and Test

**Figure 5:**
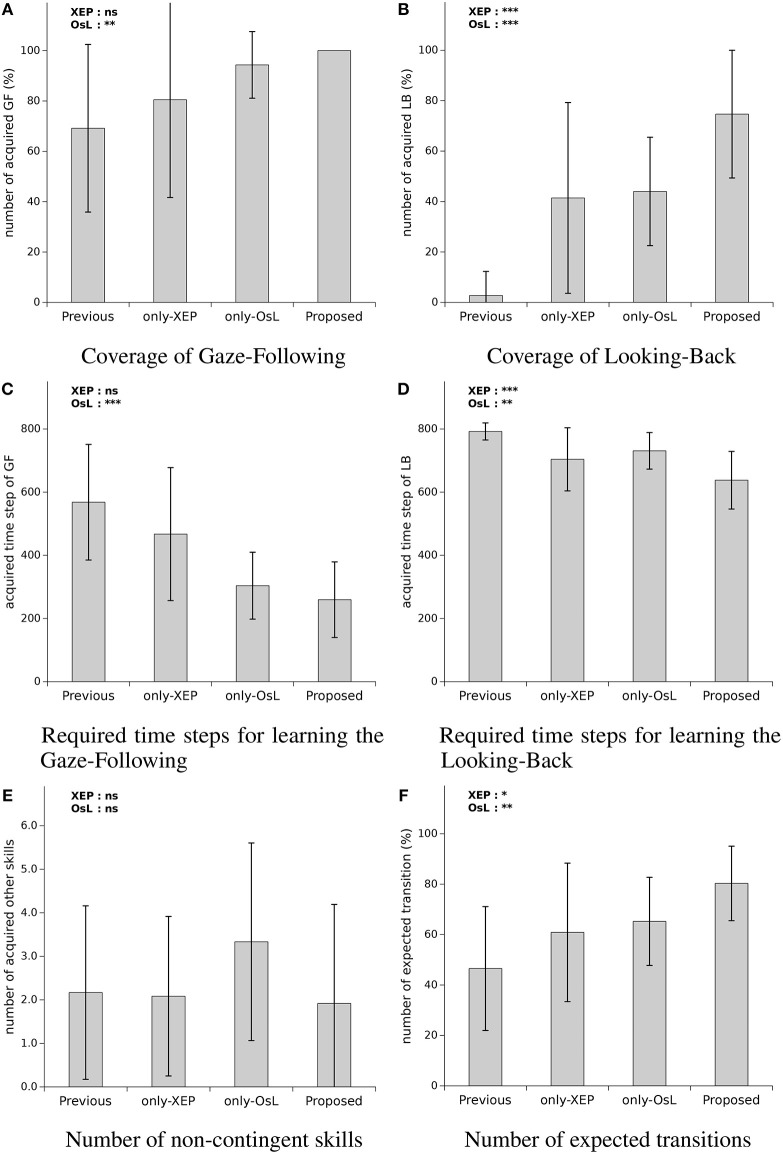
Performance comparison of the four systems: **(A)** the number of learned skills labeled Gaze-Following, **(B)** the number of learned skills labeled Looking-Back (looking back), **(C)** spent time steps to acquire Gaze-Following, **(D)** spent time steps to acquire Looking-Back, **(E)** the number of the skills which is suppose to be not contingent but acquired, and **(F)** the number of transitions where the robot succeeded in observing a result as expected by using the acquired skills. At the top left side of each graph, significant levels of main effects in two-way ANOVA with OsL (Ostensive-cue sensitive Learning) and XEP (Exclusice Evaluation of Policy) as between-subject factors are mentioned. The *p*-values are denoted by ***p < 0.001, **p < 0.01, *p < 0.05, and ns, not significant. Note that any interactions were not confirmed with the ANOVA.

The result of the performance comparison and ANOVA was summarized in Figure 5. In each graph of the figure, the average, and the standard deviation of the data gathered from the subject experiment was plotted. Additionally, the effect of each algorithm on the performance measure was denoted with the asterisk on the top left side of each figure, indicating the obtained *p*-value for the main effect of each algorithm by ANOVA^2^. The result of the mentioned two-way ANOVA for each of the performance measure is as follows.

For the coverage of Gaze-Following (Figure 5A), the ANOVA revealed a main effect of OsL, *F*_(1, 44)_ = 8.57, $p=0.005,\eta_{p}^{2}=0.163$, with 1−β = 0.846, indicating that with using the OsL algorithm the coverage of Gaze-Following was higher (*M* = 92.1%, *SD* = 9.6) than the case that the OsL was not used (*M* = 74.8%, *SD* = 35.8). The significance was not confirmed neither for the main effect of XEP *F*_(1, 44)_ = 1.24, *p* = 0.27, nor for the interaction between the OsL and XEP, *F*_(1, 44)_ = 0.14, *p* = 0.71. Note that according to Figure 5A, the coverage of Gaze-Following was 69% (*SD* = 33) using the previous method, which increased to 81% (*SD* = 39) by applying the XEP, 94% (*SD* = 13) with the OsL, and 100% (*SD* = 0) using both of them as in the proposed method. The result of ANOVA for the coverage of Looking-Back (Figure 5B) showed a main effect of OsL, *F*_(1, 44)_ = 25.4, $p<0.001,\eta_{p}^{2}=0.366$, with 1−β = 0.999, indicating that the mean coverage of Looking-Back was greater when the OsL algorithm was adopted (*M* = 59.3%, *SD* = 27.8) than the cases that the OsL was not used (*M* = 22.1%, *SD* = 33.4). Also, the main effect of XEP yielded an F ratio of *F*_(1, 44)_ = 21.7, $p<0.001,\eta_{p}^{2}=0.333$, where 1−β = 0.998, indicating that the mean coverage of Looking-Back was higher by using the XEP algorithm (*M* = 58.0%, *SD* = 35.8) than the cases that the XEP was not adopted(*M* = 23.4%, *SD* = 26.6). These main effects were not qualified by an interaction between OsL and XEP, *F*_(1, 44)_ = 0.29, *p* = 0.59. Note that as mentioned in Figure 5B, the low performance of the previous method was improved from 3% (*SD* = 10) to 75% (*SD* = 25) by using the proposed method.

For the time required to learn Gaze-Following (Figure 5C), the main effect of OsL was confirmed with the ANOVA, *F*_(1, 44)_ = 25.9, $p<0.001,\eta_{p}^{2}=0.370$, with 1−β = 0.999, indicating that the mean time required for the acquisition of Gaze-Following was faster when the OsL algorithm was adopted (*M* = 282, *SD* = 113) compared to the cases that the OsL was not used (*M* = 518, *SD* = 200). However, the significance was shown neither for the main effect of XEP, *F*_(1, 44)_ = 2.45, *p* = 0.125, nor for the interaction between the OsL and XEP, *F*_(1, 44)_ = 0.371, *p* = 0.55. Note that as mentioned in Figure 5C, the time required to learn Gaze-Following became less than the half in the proposed method compared with the previous method, i.e., decreased from 568 steps (*SD* = 183) to 260 steps (*SD* = 120).

In the case of the time required to learn Looking-Back (Figure 5D), the result of ANOVA revealed a main effect of OsL, *F*_(1, 44)_ = 8.72, $p=0.005,\eta_{p}^{2}=0.165$, with 1−β = 0.854, indicating that the mean time for the learning of the Looking-Back was faster by using OsL (*M* = 684, *SD* = 89) than not using the OsL (*M* = 748, *SD* = 85). Also, the main effect of XEP yielded an F ratio of *F*_(1, 44)_ = 17.6, $p<0.001,\eta_{p}^{2}=0.286$, where 1−β = 0.989, suggesting that the mean time required to learn Looking-Back was faster when the XEP algorithm was adopted (*M* = 670, *SD* = 100) compered with the cases which the XEP was not used (*M* = 760, *SD* = 54). The significance was not confirmed for the interaction between the OsL and XEP, *F*_(1, 44)_ = 0.013, *p* = 0.91. Note that the average time spent for the acquisition of the first Gaze-Following and Looking-Back skills by the robot using the proposed method was 8 min and 25 min with the standard deviation 5 and 7 min, respectively.

The result of the ANOVA for the number of the acquired non-contingent skills (Figure 5E) showed neither the main effect of OsL, *F*_(1, 44)_ = 0.68, *p* = 0.41, nor the main effect of XEP, *F*_(1, 44)_ = 1.53, *p* = 0.22, nor the interaction between the OsL and XEP, *F*_(1, 44)_ = 1.21, *p* = 0.28. As mentioned in the figure, when only the OsL algorithm was utilized, it increased from 2.2 (*SD* = 2.0) to 3.3 (*SD* = 2.3), while adopting the XEP decreased it to 1.9 (*SD* = 2.3) with the proposed method. However, no significant effects of either of the algorithms were found in the result of the ANOVA for this measure. Finally, for the number of the expected transition (Figure 5F), the ANOVA revealed a main effect of OsL, *F*_(1, 44)_ = 9.28, $p=0.004,\eta_{p}^{2}=0.174$ with 1−β = 0.875, indicating that the mean number of the expected transition was grater when the OsL algorithm was adopted (*M* = 72.8%, *SD* = 17.6) than the cases that the OsL was not used (*M* = 53.7%, *SD* = 26.5). Also the main effect of XEP was supported by the ANOVA, *F*_(1, 44)_ = 5.51, $p<0.023,\eta_{p}^{2}=0.111$, where 1−β = 0.669, which suggests that the mean number of the expected transition was grater by using the XEP (*M* = 70.6%, *SD* = 23.7) compared with the cases that the XEP was not implemented (*M* = 55.9%, *SD* = 22.9). It is not confirmed for the interaction between the OsL and XEP, *F*_(1, 44)_ = 0.003, *p* = 0.96. Note that according to the figure, the proposed method increased the number of the expected transition from 47% (*SD* = 25) to 80% (*SD* = 15).

#### 4.4.3. Discussion

The OsL algorithm improved the coverage of Gaze-Following while both of the XEP and OsL algorithms improved the coverage of Looking-Back. Therefore, the XEP seems to be effective on learning complex skills, such as Looking-Back, while the OsL is useful to learn both complex and simple skills, such as Gaze-Following. The reason for these are considered to be the increased accuracy of the contingency evaluation (for XEP), and synchronizing the teaching/learning phases of the caregiver/robot (for OsL). Thus, adopting both of them will lead to the highest performance in terms of the coverage of the skill acquisition. For the Gaze-Following skill, the OsL improved the time required to learn Gaze-Following. For the Looking-Back skill, both the XEP and OsL algorithms improved the time required to learn Looking-Back. The OsL seems to be effective on the time required to learn Gaze-Following and the time required to learn Looking-Back due to the synchronization problem described in section 3.1, while in the case of XEP, increasing the accuracy of the contingency evaluation, and as a result, the number of the acquired Looking-Backs seems to be the reason of the improvement. Thus, adopting both the algorithms will produce the best performance of the learning speed for the robot. The OsL uses weighted learning, which may increase the acquisition of the non-contingent skills, and the XEP may compensate it by increasing the accuracy of the contingency evaluation. However, we could not conclude anything because no significant effects of either of the algorithms and their interaction were found. Both the XEP and OsL improved the number of the expected transition. Therefore, using both of the algorithms are suggested to improve the predictability of the robot's behavior.

The most significant contribution of the current paper is building a real humanoid robot that could acquire complex social skills through sub-hour face-to-face interaction with a human while the previous work focused on the computer simulation or needed enormous interaction steps corresponding to several hours in the real world (Sumioka et al., 2010; Mugan and Kuipers, 2012; Mahzoon et al., 2016). It is worth noting that the proposed mechanism still succeeded in reproducing some infant developmental processes for social behavior resembling gaze following (Butterworth and Jarrett, 1991) and social referencing (Tomasello et al., 1995) as reported in the previous work (Sumioka et al., 2010; Mahzoon et al., 2016), although it is limited to involving the superficial similarities. Furthermore, it is also worth noting that the proposed mechanism could adapt to the behavioral changes in human, that is the emergence of a rewarding response to the behavioral changes in the robot, by extending the previously acquired skills. These features provide us with a research platform for further investigations of the flexible or variable developmental mechanism of human-like social skills in a dynamic and open-ended environment. However, since the current implementation was still limited to skills represented by combinations of several action and sensory variables, how to treat more rich variables for more complex skills will be the important future work.

### 4.5. Subjective Evaluation

#### 4.5.1. Questionnaire and Result of Test

To evaluate whether the skill acquisition processes of the robot utilizing different algorithms make a difference in the subjective opinion of the participants about the quality of the interaction as well as the feeling about the intelligence of the robot, we conducted a subjective evaluation using a questionnaire. It consisted of seven questions, which were designated with Q1–Q7. The answers were proposed as five-level Likert scale, where 5 presented strongly agree and 1 presented strongly disagree. Additionally, to evaluate the transition of the answers over time, we administered the questionnaire every 200 steps, i.e., approximately every 10 min.

**Figure 6:**
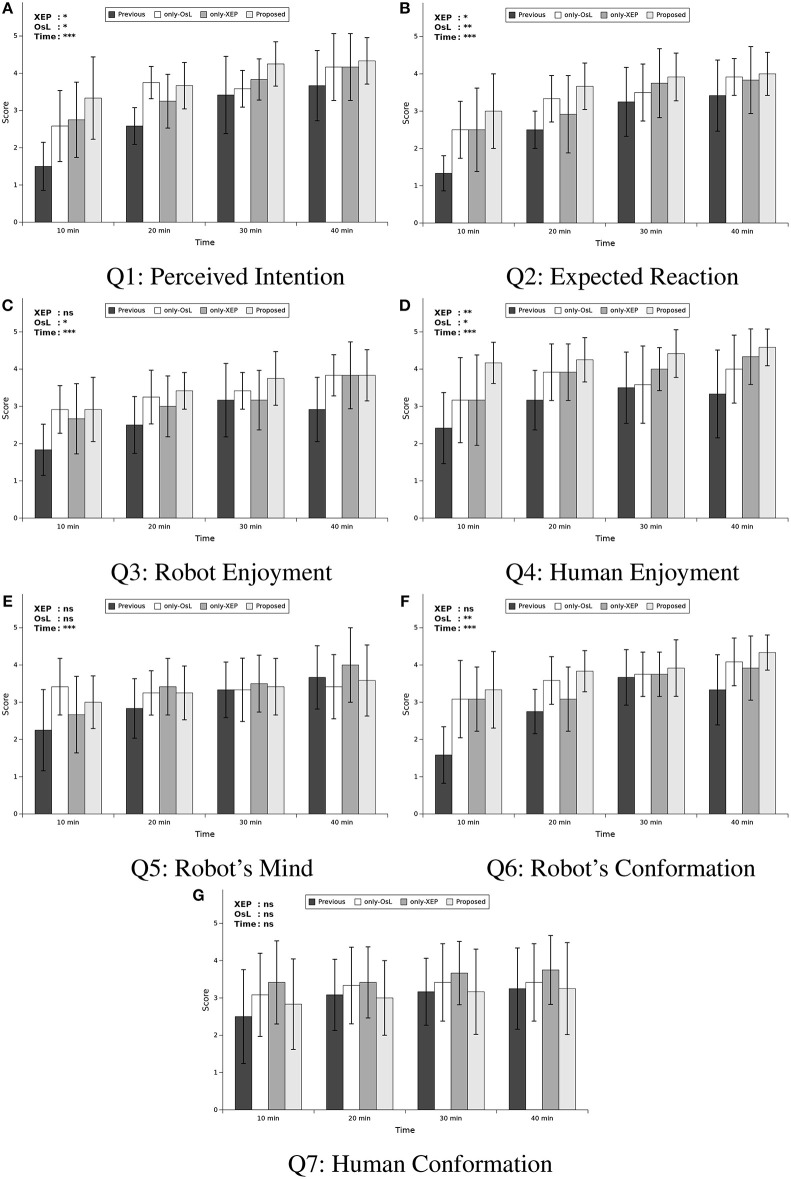
Mean scores of questionnaire. **(A)** Q1: The robot understood my intention, **(B)** Q2: The robot reacted as I expected, **(C)** Q3: The robot looks like it is enjoying the interaction, **(D)** Q4: I enjoyed the interaction, **(E)** Q5: I felt that the robot had its own mind and behaved based on it, **(F)** Q6: The robot conformed its behavior to my behavior, and **(G)** Q7: I conformed my behavior to robot's behavior. Each sub-figure includes four comparisons in each time step (*t* = 10, 20, 30, and 40 min) among four conditions of learning method: previous work, learning only with Ostensive-cue sensitive Learning (only-OsL), learning only with Exclusive Evaluation of Policies (only-XEP), and learning both with the OsL and XEP (proposed). At the top left side of each graph, significant levels of main effects in the follow-up univariate ANOVA with Time as within-factor and OsL and XEP as between-subject factors are mentioned. The *p*-values are denoted by ***p < 0.001/7, **p < 0.01/7, *p < 0.05/7, and ns, not significant, considering Bonferroni correction concerning the number of the questions, i.e., 7 question items. Note that any interactions were not confirmed with the ANOVA.

Figure 6 shows the average and standard deviation of the answers (described as score) to each question over time for each condition of the experiment. The statement used for each question is brought in the caption of the figure. A mixed-design three-way MANOVA was conducted with three independent variables (IVs) and seven dependent variables (DVs), to indicate the effect of using each algorithm (XEP and OsL) as two between subjects variables and also the course of time (hereafter denoted with “Time”) as a within subjects variable on the score of the questions (score of each questionnaire Q1–Q7) as DVs of the test. The XEP and OsL indicated whether the corresponding algorithms were used in the experiment while the Time indicated the time that the questionnaire was taken and the score was obtained, which were consisted of four levels, i.e., 10, 20, 30, and 40 min. Also, a *post-hoc* power analysis was conducted to determine the observed power (1−β) of the test, computed using α = 0.05.

The result of the test suggested a significant multivariate effect of all three IVs, XEP (Wilk's Λ = 0.677, $F_{\left(7,38\right)}=2.59,p=0.027,\eta_{p}^{2}=0.323,1-\beta =0.826$), OsL (Wilk's Λ = 0.635, $F_{\left(7,38\right)}=3.12,p=0.011,\eta_{p}^{2}=0.365,1-\beta =0.899$) and Time (Wilk's Λ = 0.179, $F_{\left(21,24\right)}=5.26,p<0.001,\eta_{p}^{2}=0.821,1-\beta =1.000$) across the DVs. However, no significant interaction was revealed in the result of the multivariate test. In the follow-up univariate ANOVAs, while several main effects were revealed, no interaction between the factors was confirmed. The result of the test was summarized in Table 5, where only the revealed significance was mentioned. In this table, for the within subjects variable Time, except of Q3 and Q5, the result of Mauchly's test indicated that the assumption of sphericity had been violated, therefore the degrees of freedom were corrected using Greenhouse-Geisser estimates of sphericity. Note that the univariate ANOVAs were conducted using Bonferroni adjusted alpha levels of .007 concerning the number of the questions, i.e., .05/7. Also, to facilitate the discussion, the result of the univariate ANOVAs were summarized in the top left side of each graphs in Figure 6, indicating the p value of the main effect for the independent variables. As shown in the figure, it was revealed that the XEP algorithm was effective to increase the score of perceived intention (Q1), expected reaction (Q2), and human enjoyment (Q4), while the OsL algorithm was also effective to increase these scores in addition to the other twos; robot enjoyment (Q3) and robot's conformation (Q6). Also, this figure and Table 5 showed that the variable Time had main effect on all DVs, except of Q7.

**Table 5:** Result of the follow-up univariate ANOVA for the questionnaire.

**Item**	**Factor**	***df*_1_**	***df*_2_**	***F*(*df*_1_, *df*_2_)**	***p***	**$\eta_{p}^{2}$**	**1−β**
Q1	XEP	1	44	10.7	0.002	0.196	0.949
	OsL	1	44	11.5	0.001	0.208	0.963
	Time	2.01	88.5	47.8	0.000	0.521	1.000
Q2	XEP	1	44	9.41	0.004	0.176	0.917
	OsL	1	44	12.0	0.001	0.215	0.969
	Time	2.53	111	38.3	0.000	0.465	1.000
Q3	OsL	1	44	10.0	0.003	0.186	0.934
	Time	3	132	24.2	0.000	0.355	1.000
Q4	XEP	1	44	14.6	0.000	0.249	0.989
	OsL	1	44	7.96	0.007	0.153	0.862
	Time	2.32	102	12.1	0.000	0.215	1.000
Q5	Time	3	132	11.8	0.000	0.211	1.000
Q6	OsL	1	44	15.2	0.000	0.256	0.992
	Time	2.04	89.7	26.7	0.000	0.378	1.000

*Only the significant factors are mentioned for each question items, considering Bonferroni adjusted alpha levels of .007 (i.e., .05/7) concerning the number of the questions, i.e., 7 questions. The degree of freedom for the factor and the error for the F-test was denoted with df_1_ and df_2_, respectively. The result of the F-test [F(df_1_, df_2_)], p-value (p), effect size ($\eta_{p}^{2}$) and the power of the test (1−β) were denoted as well*.

To indicate how the scores were changed in the course of time, the *post hoc* multiple comparison using Dunnett's method was conducted for the variable Time, using Bonferroni adjusted alpha levels of .007 concerning the number of the questions, i.e., .05/7. In this comparison, the score at Time=10min was compared with the others, i.e., Time = 20, 30, and 40min. The result of the comparison was summarized in Table 6. As shown in the table, for all of the questions mentioned in the table, the score was significantly increased from Time = 10 min to all of the other Times, except for one case, i.e.m, for Time = 20 min in Q5. In other words, it was revealed that compared to the first subjective evaluation (i.e., at Time = 10 min), the evaluation of the perceived intention (Q1), expected reaction (Q2), robot enjoyment (Q3), human enjoyment (Q4), and robot's conformation (Q6) were significantly increased after the second evaluation (i.e., at Time = 20 min), while the evaluation for robot's mind (Q5) was significantly increased after the third evaluation (i.e., at Time = 30 min).

**Table 6:** Result of the multiple comparison with Dunnett's method for the variable Time considering Bonferroni adjusted alpha levels of 0.007 (i.e., 0.05/7) concerning the number of the questions, i.e., 7 questions.

**Item**	***M*_1_**	***SD*_1_**	***T*_2_**	***M*_2_**	***SD*_2_**	**p**	**Cohen's d**	**1−β**
Q1	2.54	1.17	20	3.31	0.75	0.000	0.787	1.000
			30	3.77	0.78	0.000	1.231	1.000
			40	4.08	0.90	0.000	1.482	1.000
Q2	2.33	1.08	20	3.10	0.86	0.000	0.792	1.000
			30	3.60	0.87	0.000	1.297	1.000
			40	3.79	0.80	0.000	1.529	1.000
Q3	2.58	0.92	20	3.04	0.80	0.001	0.532	0.950
			30	3.38	0.82	0.000	0.912	1.000
			40	3.60	0.87	0.000	1.140	1.000
Q4	3.22	1.19	20	3.81	0.84	0.000	0.566	0.970
			30	3.88	0.91	0.000	0.609	0.985
			40	4.06	1.00	0.000	0.759	0.999
Q5	2.83	1.02	20	3.19	0.76	0.051	0.389	0.752
			30	3.40	0.79	0.001	0.616	0.987
			40	3.67	0.95	0.000	0.845	1.000
Q6	2.77	1.17	20	3.31	0.80	0.001	0.540	0.956
			30	3.77	0.69	0.000	1.040	1.000
			40	3.92	0.85	0.000	1.122	1.000

*In the comparison, the Time = 10 was compared with the others. In the columns of the table, the question item (Item), the mean and SD of the scores for the question at Time = 10 (M_1_ and SD_1_, respectively), the time that compared with (T_2_), the mean and SD of the scores for T_2_ (M_2_ and SD_2_, respectively), the p-value of the comparison (p), the effect size (Cohen's d) and the power of the test (1−β) were indicated*.

#### 4.5.2. Discussion

The factor of time was effective on the improvement of all of the question items, except for human conformation (Q7). The improvement of the scores from 10 to 20 or 30 min indicated that, in course of time, the robot even with neither of the proposed algorithms seemed to became looking more positive in many aspects, understanding human's intention (Q1), reacting as human expected (Q2), having its own mind (Q5), and conforming its behavior to human's behavior (Q6), and enjoying interaction (Q3) while human became enjoying interaction (Q4). This suggests that the basic developmental algorithm of the skill acquisition worked properly based on the subjective criteria.

The XEP and OsL algorithm were both effective on improving the score of some questions. Meanwhile, they improved the learning performance of skills necessary to follow the human's instruction, which is Gaze-Following and Looking-Back. Therefore, the human subjects seemed to feel that the robot understood his/her intention (Q1) of instruction, reacted as he/she expected (Q2), and consequently he/she could praise the robot more often which would make the interaction more enjoyable for the human (Q4). On the other hand, only the OsL had the main effect on the scores of robot's enjoyment (Q3) and robot conformation (Q6). It is considered to be sub-effects of the stopping behavior of the robot toward the human adopted in the OsL, which could represent the robot's attitude to positively follow the human's behavior. However, the results of the ANOVA for Q5 and Q7 had no significant effect of either of the proposed algorithms. A *post hoc* interview revealed that some subjects found negative meaning in the word “human conformation” (Q7). Also, the meaning of “mind” in Q5 might largely vary among the subjects. These might mean that they are difficult to be directly used as subjective measures.

In sum, we compared the result of the subjective evaluation of the participants in different conditions of the experiment related to their opinion about the quality of the interaction as well as the intelligence of the robot. The result showed a significant effect of the OsL and XEP algorithm on the evaluation. As described in section 1, when a caregiver recognizes a contingent and intelligent reply from an infant, he/she usually changes his/her behavior to teach a new concept to the infant. Assuming that the increase in the result of the evaluation expressing the higher level of such recognition, we can conclude that the proposed algorithms are significantly effective in inducing the caregiver to change his/her behavior and teach the infant robot a new concept. Consequently, the OsL and XEP could successfully contribute to an increase in an open-ended development of the infant robot. However, the items of the questionnaire applied in this part were not completely independent and there were correlation among some of them. Since a set of questionnaire to evaluate how the impression of the subjects about the robot was changed along with its development is not established yet, studying and inventing a suitable set with a factor analysis for such evaluation is an important future work of this field.

## 5. Conclusion

In this paper, we proposed two novel algorithms to improve the performance of the social skill learning of an infant robot during interaction with a human caregiver: namely the Ostensive-cue sensitive Learning (OsL) and the Exclusive Evaluation of Policies (XEP) algorithms. The OsL was inspired by the natural pedagogy of the human being and proposed a synchronized weighted learning mechanism based on the ostensive signals of the caregiver. The XEP algorithm proposed a way to improve the accuracy of the contingency evaluation by separating the histogram of the contingencies related to the acquired policies and atomic variables. The OsL was expected to increase the learning speed of the robot, while the XEP was expected to improve the accuracy of the contingency evaluation, especially those related to the acquired policies (i.e., complex skills).

The results of our humanoid robot experiment with human subjects showed that the OsL was effective in increasing the learning speed of the simple and complex skills, and consequently increasing the number of learned skills by the robot; while the XEP increased the accuracy of the contingency evaluation and was effective in increasing the coverage of complex skills as well as the time-steps required for the learning. These improvements resulted in enabling the infant robot and the human subject to predict each others' behavior. As a result, statistical analysis of the experiment showed a significant effect of both algorithms on increasing the number of the expected transition of the infant robot, the subjective evaluation of the human participants about the quality of the interaction and the intelligence of the robot. Since the level of the recognition of the human caregiver about the intelligence of the robot has an impact on the teaching tendency of the caregiver, the increase in the subjective evaluation can be expressed as a contribution of the proposed algorithms on increasing the opportunity of the open-ended development of the infant robot. Finally, the proposed mechanism of this paper enabled the robot to learn some primitive social skills *within a short time-step of a real-world interaction* with a human subject: simple skills such as the Gaze-Following behavior after 8 min, and complex skills such as Looking-Back behavior after 25 min.

However, the variables utilized in this work were assumed to be quantized, and the modality of the sensory and action variables of the robot were still few. Utilizing dynamic quantization methods such as that proposed in the previous work (Mugan and Kuipers, 2012) could be a way to treat continuous variables. Meanwhile, the way to dynamically adapt the learning parameters of the system to the developmental change in quantization level would be an important topic. Research on this topic will propose an insight about the developmental models, which may be compared with the model of human infant. Moreover, adding more modalities to the variables, such as the voice of the caregiver to the sensory variables, and speaking/uttering ability to the action variables of the robot could increase the complexity of the interaction as well as that of acquired skills by the robot. Nevertheless, treating with the probable huge varieties of the caregiver's behavior will be one of the challenging issues for the implementation of the developmental robot in such an environment. These problems are needed to be considered as the main topics of the future work.

## Author Contributions

HM wrote computer code, performed the modeling and subject experiment, analyzed output data, and wrote the paper manuscript. YY supervised the modeling, experimental setup, subject experiment, and data analyses. HI supervised the project.

### Conflict of Interest Statement

The authors declare that the research was conducted in the absence of any commercial or financial relationships that could be construed as a potential conflict of interest.

## Footnotes

^1^For the same reason, the system also uses the *extra* variables ${\hat{S}}_{i}^{t}$ and ${\hat{A}}_{j}^{t}$ when the robot has used the policy in the previous time step, i.e., when *S*_π_*m*__ = 1.

^2^The *p*-values are denoted by ^***^*p* < 0.001, ^**^*p* < 0.01, ^*^*p* < 0.05, and ns, not significant, in the figures of this paper.
